# Urologically silent intravesical migration of a copper intrauterine device presenting as chronic pelvic pain: CT features and comparison with reported cases

**DOI:** 10.1016/j.radcr.2026.09.084

**Published:** 2026-10-19

**Authors:** Montacer Hafsi, Asma Zouaghi, Oumayma Yaich, Azer Hafsi, Safa Azzouzi, Nejmeddine Jellali

**Affiliations:** Department of Gynecology and Obstetrics, Kasserine Maternity Center, Université Of Sousse, Kasserine, Tunisia

**Keywords:** Intrauterine device, Uterine perforation, Urinary bladder, Foreign-body migration, Computed tomography, Cystoscopy

## Abstract

Uterine perforation with migration of an intrauterine device (IUD) into the urinary bladder is a well-documented complication that usually presents with lower urinary tract symptoms, recurrent urinary tract infection, hematuria, or a secondary bladder calculus. We report a 35-year-old woman (G2P2) with a copper IUD inserted 5 years earlier who presented with progressive chronic pelvic pain and no urinary symptoms. Pelvic ultrasound showed an abnormally positioned device, and contrast-enhanced computed tomography (CT) demonstrated a T-shaped device of metallic density traversing the anterior uterine wall, with one arm protruding into the bladder dome and coated by a small encrustation. The fixed, non-dependent position of the linear metallic core, its continuity with the myometrium, and its T-shaped morphology distinguished the device from a primary bladder calculus. Cystoscopy confirmed partial intravesical protrusion of the encrusted arm, which was extracted intact with forceps without complication, and the patient was asymptomatic at follow-up. Compared with published intravesical cases, in which urinary complaints or a bladder stone usually prompt investigation, this case shows that bladder involvement may remain silent from a urinary perspective. In IUD users with unexplained pelvic pain, imaging should specifically assess the bladder, and a delayed excretory CT phase is advisable to evaluate bladder-wall integrity before removal.

## Introduction

The intrauterine device (IUD) is one of the most effective and widely used methods of long-acting reversible contraception. Uterine perforation is uncommon: in a large European prospective cohort it occurred in approximately 1.3 per 1000 insertions, with a markedly higher risk when insertion took place during breastfeeding or within 36 weeks after delivery [1,2]. Perforation may be complete or partial, may occur at insertion or be recognized only later, and allows the device to migrate into the peritoneal cavity or into adjacent organs [[2], [3], [4]].

Migration into the urinary bladder is well documented. Since the classic reviews of Zakin et al. [4], numerous case reports and series, including 2 Tunisian series of 6 and 10 patients, have described intravesical IUDs; most presented with lower urinary tract symptoms (LUTS), recurrent urinary tract infection, hematuria, or a secondary bladder calculus [[5], [6], [7], [8], [9]]. Asymptomatic or minimally symptomatic extrauterine devices have also been reported [10,11]. The present case is therefore not reported as a rare or novel entity.

Our aim is educational. We describe a partial intravesical migration of a copper IUD in a woman whose only complaint was chronic pelvic pain, detail the computed tomography (CT) features that allowed the device to be distinguished from a primary bladder calculus and other pelvic calcifications, and compare the presentation with previously reported intravesical cases (Table 1). The practical message is that intravesical migration may remain silent from a urinary perspective and can therefore escape a urologically oriented work-up.

**Table 1:** Presentation and management of the present case compared with representative published cases of intravesical IUD migration.

Study	Age (y) / time since insertion	Presenting complaint	Urinary symptoms	Imaging	Findings	Management / outcome
Demirci et al., 2003 [8]	33 / 2 y	Irritative LUTS	Yes	Plain radiography	Copper T with large bladder stones	Cystoscopic electrohydraulic lithotripsy and forceps extraction; uneventful
Sallami et al., 2011 [5] (10 patients, Tunisia)	NR / 2–12 y to symptoms	Persistent LUTS in almost all; gross hematuria in 4	Yes	Ultrasound and/or IV urography	Stone in all; device partially intravesical in 9	Endoscopic lithotripsy and extraction; drainage 10 days; uneventful at 2 y
De Silva et al., 2017 [9]	48 / 15 y	LUTS and recurrent UTI	Yes	Plain radiography, ultrasound	Large laminated stone with IUD imprint	Open vesicolithotomy; asymptomatic
Houmaid et al., 2024 [7]	35 / 5 y	Chronic pelvic pain with burning micturition, frequency, dysuria	Yes	Ultrasound, plain radiography	IUD embedded in bladder wall with calcification; urine culture negative	Cystoscopic removal; symptom-free at 1 month
Gupta et al., 2024 [11]	32 / 7 y	Pregnancy (contraceptive failure)	No	Imaging (see ref.)	Encrusted IUD, no fistula	Cystoscopic fragmentation and retrieval
Present case	35 / 5 y	Isolated chronic pelvic pain	No	Ultrasound, contrast-enhanced CT	Transmural device; one encrusted arm protruding into bladder dome	Cystoscopic forceps extraction; catheter 10 days; asymptomatic at 6 weeks

CT, computed tomography; IUD, intrauterine device; IV, intravenous; LUTS, lower urinary tract symptoms; NR, not reported; UTI, urinary tract infection. Nouira et al. [6] reported 6 further Tunisian patients with intravesical IUDs complicated by stones, all managed endoscopically.

## Case presentation

A 35-year-old woman, gravida 2 para 2 (mode of delivery: vaginal/cesarean), presented to our gynecology department with a [duration]-month history of progressively worsening chronic pelvic pain, predominantly suprapubic and [non-cyclical/cyclical]. A copper T-shaped IUD ([model, e.g., TCu380A]) had been inserted 5 years earlier for contraception ([interval insertion / x weeks postpartum; breastfeeding at insertion: yes/no]). The insertion was reportedly uneventful, although she could not recall details of the provider’s experience or of any technical difficulty. No follow-up visit or thread check had been performed after insertion. The device was within its licensed duration of use and was being used for ongoing contraception; removal was indicated by malposition rather than by expiry of the device [12].

She denied dysuria, hematuria, urinary frequency, and recurrent urinary tract infections [confirm: no urgency, no leakage of urine per vagina]. Her menstrual cycles were regular. Her medical and surgical history was unremarkable. She was afebrile and hemodynamically stable. Abdominal examination revealed mild suprapubic tenderness without guarding or peritoneal signs. On speculum examination, the IUD strings were not visible at the external os. Bimanual examination showed [uterine size, position, tenderness, and there was no vaginal leakage of urine].

Urinalysis dipstick: leukocytes, nitrites, hematuria and urine culture [sterile] were obtained. The complete blood count and C-reactive protein were [values], and the urine pregnancy test was negative. A plain abdominopelvic radiograph (kidney–ureter–bladder) [showed a radiopaque T-shaped device projecting over the bladder].

Transvaginal [and transabdominal] ultrasound showed a normal-sized, anteverted uterus with an eccentrically located, echogenic IUD: the stem was partly within the endometrial cavity and embedded in the anterior myometrium, and one arm extended through the anterior myometrium toward the bladder dome, raising suspicion of partial migration beyond the uterus.

Contrast-enhanced CT of the abdomen and pelvis was then performed on a slice scanner after intravenous injection of [100] mL of iodinated contrast medium agent. Images were acquired in the [portal venous] phase ([60] s after injection) and reconstructed at [slice thickness] mm with sagittal and coronal multiplanar reformations. A delayed excretory phase was acquired at 5 min.

CT showed a T-shaped linear device of metallic density. Its stem lay partly within the uterine cavity within the anterior myometrium, whereas one arm traversed the anterior uterine wall and the uterovesical space and protruded into the bladder lumen at the dome. The intravesical tip was coated by a small hyperdense encrustation (Fig. 1A–B). Sagittal reformations confirmed the continuity of the device from the uterus into the bladder dome (Figs. 1C–D). There was [no free intraperitoneal fluid or air, no pelvic collection, no bowel involvement, and no intravesical gas].

**Fig. 1:**
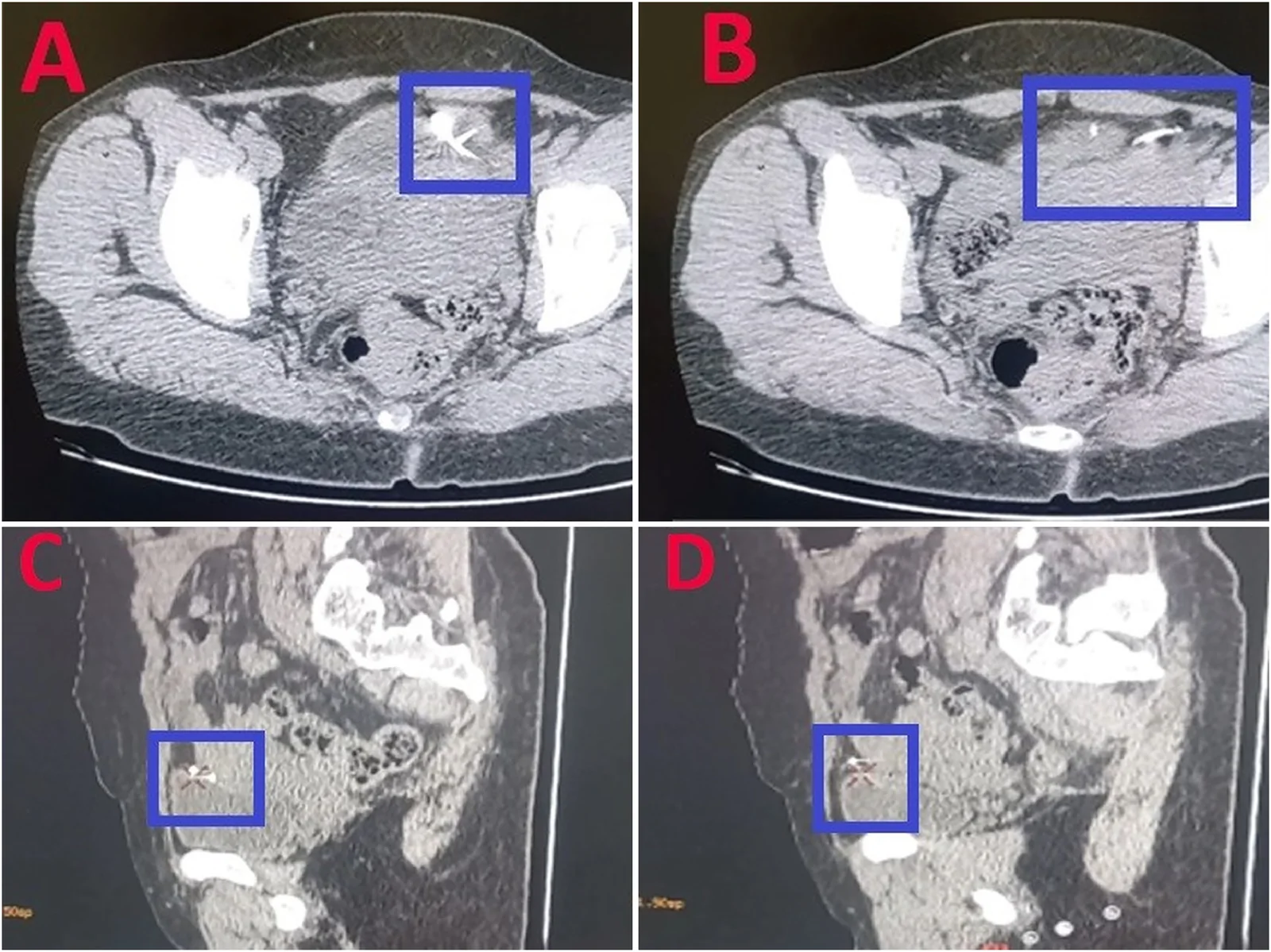
Contrast-enhanced CT of the pelvis ([phase]). (A, B) Axial images showing the T-shaped device of metallic density (box) traversing the anterior uterine wall, with one arm protruding into the bladder dome and a small hyperdense encrustation at its tip. (C, D) Sagittal reformations showing the continuity of the device from the uterus into the bladder dome (box).

Because the device was only partially intravesical, the encrustation was small, and no extravesical collection was suspected, an endoscopic approach was chosen [jointly with the urology team]. Under general anesthesia, cystoscopy ([rigid, 14Fr]) showed one arm of the IUD protruding into the bladder lumen at the dome, encrusted and surrounded by an inflammatory mucosal reaction (Fig. 2A); the remaining bladder mucosa and both ureteral orifices were [normal]. The device was grasped with foreign-body forceps and extracted intact through the urethra under direct vision, without lithotripsy (Fig. 2B). After extraction, inspection of the perforation site showed no fistulous orifice or active bleeding, and [bladder distension with saline produced no leakage through the cervix or vagina]. No intraoperative complication occurred. The retrieved device was a complete copper T-shaped IUD with calcified encrustation at the tip of one arm (Fig. 3).

**Fig. 2:**
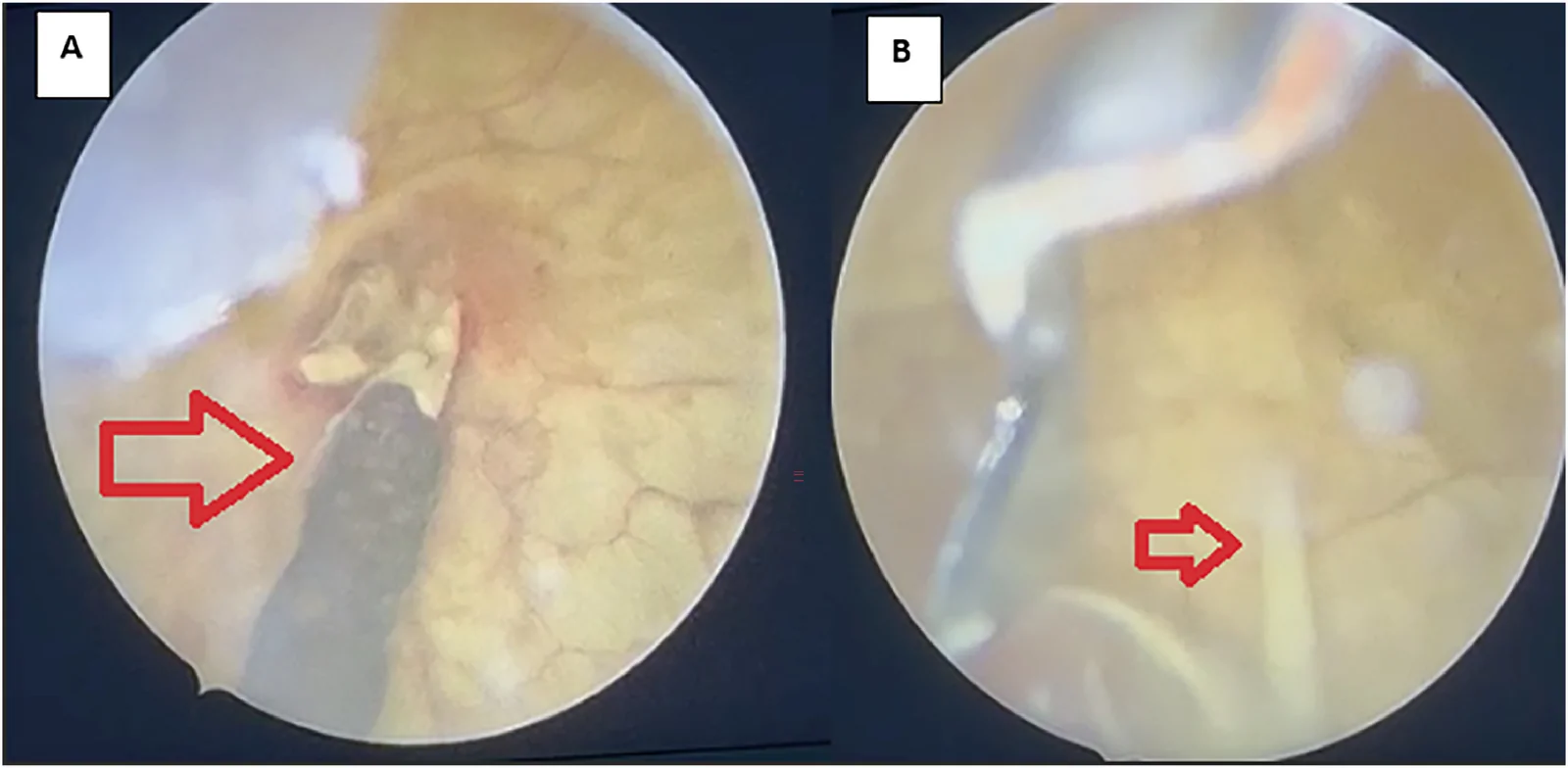
Cystoscopic views. (A) Encrusted arm of the IUD protruding into the bladder lumen at the dome (arrow), with surrounding mucosal inflammatory reaction. (B) Closer view during extraction with foreign-body forceps (arrow: device). No fistulous orifice or active bleeding was identified at the perforation site.

**Fig. 3:**
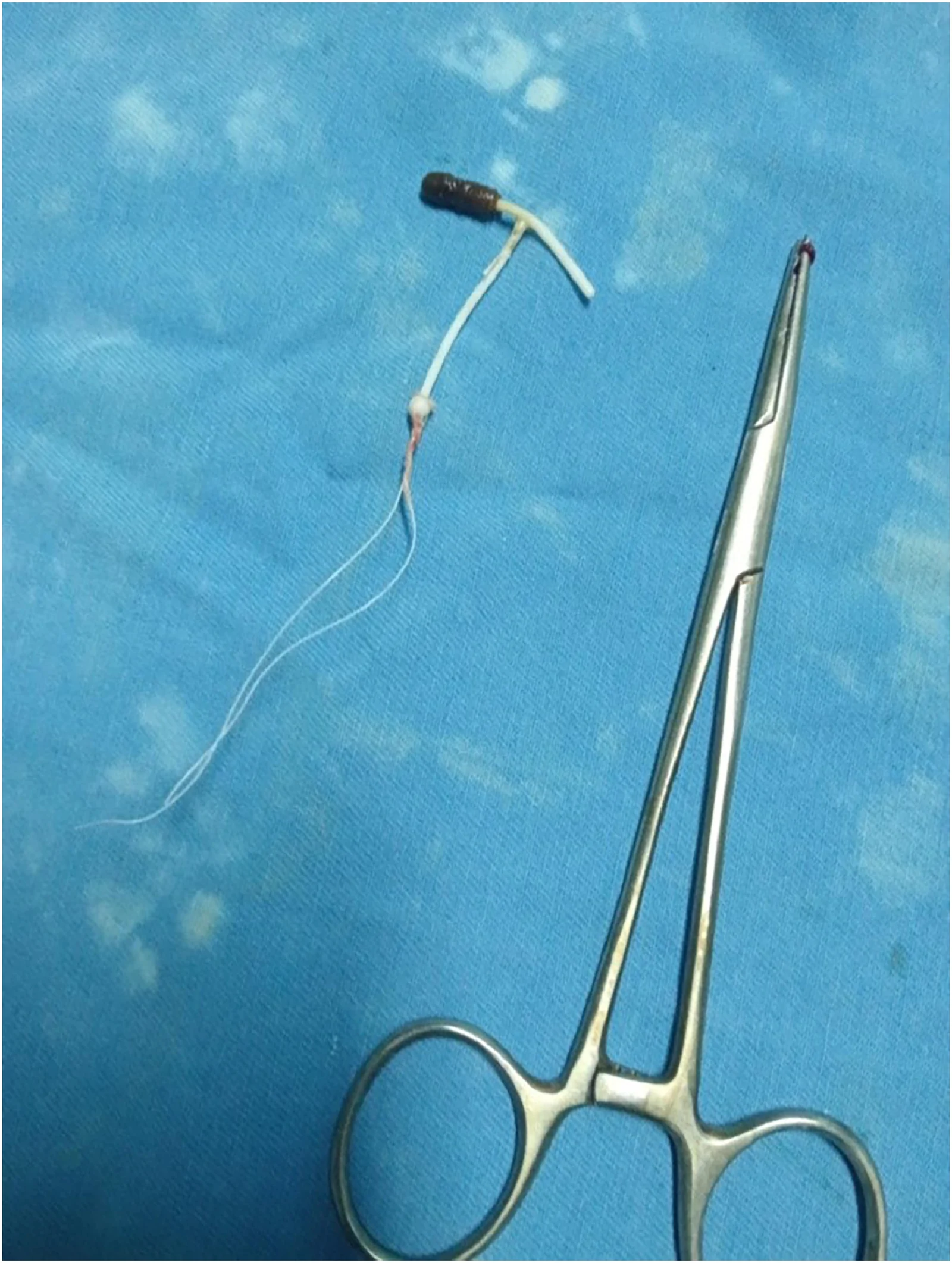
Retrieved copper T-shaped IUD showing calcified encrustation at the tip of the arm that had protruded into the bladder.

A [16/18] Fr urethral Foley catheter was left in place for 10 days to allow healing of the bladder-wall defect and to reduce the risk of vesicouterine fistula, and prophylactic antibiotics were administered. The patient was discharged on postoperative day 1 with the catheter in place. The catheter was removed on day 10 after a normal retrograde cystogram. [spontaneous voiding resumed without urinary leakage or infection]. [Alternative contraception was discussed]. At the 6-week visit, she was asymptomatic, with complete resolution of pelvic pain and no urinary complaints. At the latest follow-up, 4months after removal, she remained asymptomatic, and pelvic ultrasound was normal.

## Discussion

Intravesical IUD migration is well established, and the absence of urinary symptoms is not in itself new: minimally symptomatic and incidentally detected extrauterine devices have been described since the early reviews of IUD perforation [4,10,11]. The educational value of this case lies in 3 points: how a urologically silent presentation directed the work-up toward gynecological rather than urinary causes, the CT features that allowed a confident preoperative diagnosis and its distinction from mimics, and the implications for imaging technique and management.

Table 1 summarizes representative published intravesical cases. In the 2 Tunisian series, persistent LUTS were the leading complaint in almost all patients, macroscopic hematuria occurred in 4 of 10, the interval between insertion and symptoms ranged from 2 to 12 years, and every patient had a calculus around a device that was most often only partially intravesical [5,6]. Single reports similarly describe irritative LUTS with large stones 2 years after insertion [8], or LUTS with recurrent infection and a large laminated stone 15 years after insertion [9]. The published case most comparable to ours, a 35-year-old woman whose IUD had been inserted 5 years earlier and who presented with chronic pelvic pain, also had burning micturition, frequency, and dysuria [7]. When urinary symptoms were absent, diagnosis was usually triggered by an unrelated event such as contraceptive failure [11]. Our patient differs in that isolated pelvic pain was the only complaint, the encrustation was small rather than a bulky calculus, and the diagnosis was established by the gynecology team on cross-sectional imaging rather than during a urinary work-up.

A plausible explanation for the absence of urinary symptoms is the limited intravesical exposure: only the tip of one arm protruded into the lumen, at the dome, remote from the trigone, and the encrustation was small. The pelvic pain more likely reflected the transmural course of the device through the myometrium and the perivesical inflammatory reaction. This hypothesis cannot be proven from a single case, but it illustrates why a urinary history alone cannot exclude bladder involvement.

Ultrasound is the first-line test for locating an IUD and can show an eccentric or embedded device, but the extrauterine segment may be difficult to follow [3]. A plain radiograph confirms the presence of a radiopaque device when the strings are missing and may show a laminated stone around a T-shaped nidus, but it cannot establish the relationship of the device with the uterus or bladder wall [3,4,9]. CT with multiplanar reformations depicts the whole course of the device across the myometrium and uterovesical space, as well as associated complications [3].

An intravesical calcified focus in a woman may correspond to a primary bladder calculus, an encrusted foreign body (IUD, forgotten ureteral stent, suture, or catheter fragment), dystrophic bladder-wall calcification (schistosomiasis, tuberculosis, encrusted cystitis), or a calcified bladder tumor; extravesical mimics include a calcified leiomyoma, mature ovarian teratoma, pelvic phleboliths, and distal ureteral calculi. In our patient, 4 features favored an encrusted IUD over a primary calculus: a linear, T-shaped core of metallic density reproducing the shape of the device; continuity of this core with the anterior myometrium on sagittal reformations; a fixed, non-dependent position at the bladder dome, whereas free calculi are mobile and settle in the dependent bladder base; and a thin encrustation limited to the tip of one arm rather than a round, laminated stone. Misdiagnosis of an encrusted IUD as a primary bladder stone on imaging has been reported [13]. When doubt persists, prone or decubitus acquisition demonstrates the mobility of a free calculus.

Because transmural migration may be associated with a bladder-wall defect or uterovesical fistula, the optimal CT protocol includes a delayed excretory phase (CT urography) or CT cystography with retrograde bladder filling, which opacifies the bladder lumen and can show contrast leakage through the wall or into the uterus. [In our patient, only a portal venous phase was acquired, which is a limitation.] Fistula was therefore excluded clinically (no leakage of urine per vagina), cystoscopically (no fistulous orifice after extraction), and [by a normal cystogram before catheter removal / by the uneventful course after catheter removal].

Endoscopic retrieval is the preferred first-line option for intravesical devices and was successful in all patients of the Tunisian series, often combined with lithotripsy of the associated stone [5,6,8,11]. Open vesicolithotomy has been used for very large stones [9], and laparoscopy is preferred when the device lies predominantly in the peritoneal cavity or is densely embedded [14]. Postoperative urethral drainage for about 10 days, as in our patient, was also used in the Sallami series [5], although its optimal duration is not standardized. The device had been in place for 5 years, within the licensed duration of copper T-shaped IUDs [12]; the indication for removal was therefore malposition, not device expiry, and counseling on alternative contraception was required.

Current guidance does not support routine ultrasound after IUD insertion. The 2024 US Selected Practice Recommendations state that no routine follow-up visit is required after IUD placement and suggest that clinicians consider checking for the strings at other visits [15]. The Faculty of Sexual and Reproductive Healthcare recommends teaching users to check their threads and arranging ultrasound when threads are not felt or perforation is suspected [12]. We therefore do not advocate routine early imaging; rather, this case supports targeted ultrasound in IUD users with non-visible strings or new pelvic pain, with specific assessment of the anterior myometrium and bladder.

This report has limitations. It describes a single patient, and details of the original insertion were unavailable. [A delayed excretory CT phase was not acquired, and] the CT figures were exported from the picture archiving and communication system [confirm]. Follow-up is limited to [__ months].

## Conclusion

Intravesical IUD migration is a known complication, but it may remain silent from a urinary perspective and present only with chronic pelvic pain. In IUD users with unexplained pelvic pain, imaging should specifically evaluate the bladder. On CT, a fixed T-shaped core of metallic density in continuity with the myometrium distinguishes an encrusted device from a primary bladder calculus, and a delayed excretory phase is advisable to assess bladder-wall integrity. Cystoscopic retrieval is effective when the device is only partially intravesical.

## Ethical approval

Ethical approval was not required for this single-patient case report, in accordance with institutional policy. The patient was managed in accordance with the Declaration of Helsinki.

## Declaration of generative AI and AI-assisted technologies in the writing process

None.

## Patient consent

Written informed consent was obtained from the patient for publication of this case report and the accompanying clinical information and images. The patient reviewed the manuscript and provided consent for publication. All efforts have been made to protect the patient's identity, and no identifying personal information is included in this report.
